# Gross rainfall amount and maximum rainfall intensity in 60-minute influence on interception loss of shrubs: a 10-year observation in the Tengger Desert

**DOI:** 10.1038/srep26030

**Published:** 2016-05-17

**Authors:** Zhi-Shan Zhang, Yang Zhao, Xin-Rong Li, Lei Huang, Hui-Juan Tan

**Affiliations:** 1 Shapotou Desert Research and Experimental Station, Cold and Arid Regions Environmental and Engineering Research Institute, Chinese Academy of Sciences, 320 Donggang West Road, Lanzhou, 730000, China

## Abstract

In water-limited regions, rainfall interception is influenced by rainfall properties and crown characteristics. Rainfall properties, aside from gross rainfall amount and duration (GR and RD), maximum rainfall intensity and rainless gap (RG), within rain events may heavily affect throughfall and interception by plants. From 2004 to 2014 (except for 2007), individual shrubs of *Caragana korshinskii* and *Artemisia ordosica* were selected to measure throughfall during 210 rain events. Various rainfall properties were auto-measured and crown characteristics, i.e., height, branch and leaf area index, crown area and volume of two shrubs were also measured. The relative interceptions of *C. korshinskii* and *A. ordosica* were 29.1% and 17.1%, respectively. Rainfall properties have more contributions than crown characteristics to throughfall and interception of shrubs. Throughfall and interception of shrubs can be explained by GR, RI_60_ (maximum rainfall intensities during 60 min), RD and RG in deceasing importance. However, relative throughfall and interception of two shrubs have different responses to rainfall properties and crown characteristics, those of *C. korshinskii* were closely related to rainfall properties, while those of *A. ordosica* were more dependent on crown characteristics. We highlight long-term monitoring is very necessary to determine the relationships between throughfall and interception with crown characteristics.

Arid and semi-arid areas usually face water scarcity and conflicts for its use, thus a complete understanding of the water balance in these regions is urgently needed^1^,^2^. Rainfall interception is the process by which gross rainfall falling onto vegetative surfaces is subsequently redistributed and evaporated into the atmosphere, thus it is generally considered as a loss in the water balance, particularly in arid and semi-arid regions^3^. Therefore, rainfall interception is a vital important hydrological process in water resource management^4^. In addition, canopy interception produces significant influences on the various patterns and processes of the ecosystems in these regions, where water is always a dominant factor^5^. Therefore, more studies regarding longer-term canopy interception losses in the field are required in arid and semi-arid environments^6^,^7^.

Rainfall interception is influenced by many factors, which are generally classified into two categories, i.e. climatic factors during rain event, and structural characteristics of vegetation^7^,^8^. Rainfall properties, especially the amount, duration and intensity of rainfall have often been used to interpret field measurements of rainfall interception loss by plants in arid and semi-arid regions^6^,^9^,^10^,^11^,^12^. Recently, researchers have realized that some rainfall properties, such as maximum intensity and rainless gap within a rainfall event other than conventional parameters, can provide powerful explanations for canopy interception in arid and semi-arid regions^13^,^14^. Dunkerley^13^ held that the actual durations and intensities of rainfall bursts within each hour or other rainfall tallying period provided more information hydrologically and erosionally than mean rainfall intensity. For example, the maximum 10 and 30 min intensities (I_10_ and I_30_) have been found to offer greater explanatory power in soil erosion studies than mean rainfall intensity^15^,^16^. The intensity bursts within a rainfall event have been used to describe rainfall intensity profiles^17^, in turn showing influence on environmental processes^1^,^18^. The effects of rainfall intensity bursts on some surface processes have been reported, but much less attention has been paid to influence on canopy interception.

Furthermore, in arid and semi-arid regions there are often rainless breaks during rainfall. The period of rainless gap within a rainfall event may account for a larger proportion of the event duration than the periods of rain^6^,^19^,^20^. Rainless gap may allow wet plant canopies to partially dry, thereby re-developing the canopy storage capacity and increasing canopy storage and perhaps interception losses^3^. In addition, rainless gap may increase the time required for the canopy to becoming fully re-wetted, and hence reducing the time through which intra-rain evaporative losses can proceed at the maximum rate^21^. However, there have been few studies in which any form of rainless gap was used to explore the relationship between it and canopy interception at the plot or field scale^14^.

Crown characteristics also influence on interception loss. For an individual tree, the crown size or volume, leaf area, branching pattern have all been shown to influence rainfall interception^22^,^23^. A clear reduction in relative throughfall by trees and shrubs corresponded with increasing diameter at breast height, plant height, basal area, and leaf area index under Mediterranean conditions^24^. On the contrary, the interception loss by vegetation would certainly increase with increased canopy size^5^,^24^. However, many studies showed the influences of crown characteristics on throughfall or interception are generally less important comparing to rainfall properties in arid and semi-arid regions^5^,^22^,^23^,^24^, partly ascribing to sparse crown structure and low leaf area index of xeric shrub^11^. This must be verified using long-term observation in the field.

In the present study, the interception loss by revegetated shrubs of *Caragana korshinskii* (I_C_) and *Artemisia ordosica* (I_A_) were measured from 2004 to 2014 (except for 2007). Rainfall properties, such as gross rainfall (GR), rainfall duration (RD), maximum rainfall intensities (i.e. the maximum 10, 30, and 60 min intensities defined as RI_10_, RI_30_ and RI_60_) and mean intensity (RI), and the period of rainless gap (RG), and crown characteristics, such as plant height (H), branch and leaf area index (BLAI), crown area (C_A_) and crown volume (C_V_) of two shrub species were observed simultaneously on the southeast edge of the Tengger Desert, northern China. The aim of this case study was to determine the relationship between throughfall across the plant crown and interception by the plant crown with rainfall properties and crown characteristics, especially the impacts of maximum rainfall intensities (i.e., RI_10_, RI_30_ and RI_60_) and RG within a rain event, and to choose more appropriate parameters for a canopy interception model. We hypothesized 1) rainfall properties have more significant impacts than crown characteristics on throughfall and interception of shrubs; and 2) maximum intensities and RG have significant impacts on the throughfall and interception of shrubs.

## Materials and Methods

### Study site

The study was conducted at the Shapotou Desert Research and Experimental Station, which is located on the southeast edge of the Tengger Desert, northern China (37°33′ N, 105°02′ E) at an elevation of 1,250 m above mean sea level. The area is a typical transitional zone between desert and desert oasis. The natural vegetation is dominated by *Hedysarum scoparium* and *Agriophyllum squarrosum*, with a cover of approximately 1%. Most of the area is covered by high, dense and continuous reticulate barchan dunes. The soil substrate is loose and impoverished moving sand, with a moisture gravimetric content of 3–4%. The mean annual air temperature is 9.6 °C, extreme minimum temperature is −25.1 °C, and maximum temperature is 38.1 °C. The mean annual rainfall is 186.2 mm, with rainfall occurring mainly from July to September. The mean annual wind velocity is 2.9 m s^−1^, and dust events are recorded on an average of 122 days per year. In order to extend the research to water cycles in the restored vegetation area, the Water Balance Experimental Fields (WBEF), which occupy 10,000 m^2^ and were revegetated with *C. korshinskii* and *A. ordosica*, were established by the Shapotou Station in April 1989. The revegetation in this system plays a vital role in soil rehabilitation and production of this desert ecosystem by stabilizing dune surfaces, thus preventing wind erosion, and stabilizing the local desert ecosystem. After 15 years of environmental change, a homogeneous cover of biological soil crust now dominates the space between shrubs^25^.

## Methods

The studies were conducted in monospecific stands of *C. korshinskii* and *A. ordosica*in the WBEF from 2004 to 2014 (except for 2007). In each plot, an individual shrub was selected to measure the throughfall. Under the canopy of each plant, nine throughfall collection cups were placed. Throughfall collection cups with an inner diameter of 8.2 cm were used during 2004–2006, while cups with an inner diameter of 10 cm were used since 2008. The cups were placed beneath the shrub canopy in three rows set 120° apart, serving as three replications. For *A. ordosica*, the three farthest cups were placed 0.50 m from the stem (close to the crown edge), the three innermost cups were placed 0.10 m from the stem (close to the stem), and the three middle cups were placed 0.25 m from the stem (between the crown and edge). The cup positions for *C. korshinskii* were 0.20 m, 0.50 m and 1.00 m (Fig. S1). This layout of the cups was used to decrease the wind impact on the throughfall sampling. In order to avoid evaporative losses from the open cups, the throughfall was measured immediately after each rain event. Previous research in this area showed that the throughfall beneath the crown of *A. ordosica* and *C. korshinskii* was zero and could not be measured during an individual rainfall event with *GR* ≤ 0.5 mm^11^. Therefore, we measured the throughfall if the individual rainfall event had *GR* > 0.5 mm. The rainfall amount and duration of an individual rainfall event were each measured for 10 min by a Vaisala RG-13 tipping-bucket rain gauge, part of the Milos 520 data collection and processing system (Vaisala Automatic Weather Station, Helsinki, Finland), with an observational error of ≤±0.2 mm when GR < 10 mm, and ≤±0.02 mm when GR ≥ 10 mm. In the present study, an individual rainfall event was defined as a period or periods of rain separated from an earlier rainfall event by more than 12 h, due to sufficient time between events for the plant canopy to dry, thereby re-establishing the full canopy storage capacity prior to the commencement of rain^26^. The period of rainless gap (RG) is rainfall amount <0.1 mm h^−1^, as the rainfall amount is less than typical wet-canopy evaporation rates during rainfall^14^,^27^.

Every half month during the growing season (from April to October), the morphological characteristics of the shrubs were quantified. Plant height was measured at the center of the crown. The diameters from east to west and from north to south through the center of the fullest part of the crown were measured. C_A_ was calculated as an ellipse^28^ and C_V_ was calculated as a spheroid using plant height and crown diameters^23^. BLAI was measured beneath the crown using LAI-2000 plant canopy analyzer (Li-Cor, Inc., USA), and the measurement was taken three times in three directions (replications), about 120° apart at positions 15 cm from stem for *A. ordosica* and 30 cm for *C. korshinskii*.

### Data processing

The throughfall across the entire crown of *C. korshinskii* (T_C_) and *A. ordosica* (T_A_) was calculated using a weighting method: the weight proportions to the area of each annulus were 0.04, 0.21 and 0.75 for the innermost, middle and farthest cups, respectively, which is consistent with the collecting areas they each represent^11^. The interception losses by the whole crown of *C. korshinskii* (I_C_) and *A. ordosica* (I_A_) were obtained by subtraction of the throughfall from the gross rainfall amount (GR) over the same area, and the stemflow was omitted in the interception loss calculation, due to the fact that the crown structures of the two shrubs did not favor the production of stemflow^11^. The percentage of throughfall to GR (T_C_/GR and T_A_/GR) and the ratios of interception to GR (I_C_/GR and I_A_/GR) were calculated as T_C_ and T_A_ or I_C_ and I_A_ divided by GR. In order to describe the properties of the rainfall intensity, we used RI_10_, RI_30_ and RI_60_, the stage maximum intensities of any 10, 30 and 60 min period within the event; and RI (mean rainfall intensity) was also calculated as GR divided by rainfall duration (RD).

To analyze the effects of rainfall properties on throughfall and interception of the entire crown, we used T_C_, T_A_, I_C_, I_A_, T_C_/GR, T_A_/GR, I_C_/GR and I_A_/GR as dependent variables and GR, RD, RI_10_, RI_30_, RI_60_, RI, RG, RG/RD, H, BLAI, C_A_ and C_V_ as independent variables, and the relationships between them were fitted by linear, logarithmic or exponential functions. In addition, linear stepwise regression was conducted to analyze the relationships between T_C_, T_A_, I_C_, I_A_, T_C_/GR, T_A_/GR, I_C_/GR and I_A_/GR with GR, RD, RI_60_, RG, H, BLAI, C_A_ and C_V_. The best model to predict dependent variables was selected based on the determination coefficient (R^2^) and Akaike information criterion (AIC), which is a penalized likelihood criterion, and the best statistical model minimized the value of AIC^29^. All of the regression analyses were performed using the SPSS 16.0 statistical software (SPSS Inc., Chicago, IL, USA).

## Results

### General properties of rainfall, throughfall and interception during the experiment period

From 2004 to 2014 (except for 2007), the rainfall amounts in 2011 and 2012 were level year, approaching the multi-year mean value of 186.2 mm. The 2014 was a high flow year, with a rainfall amount 224.6 mm, and the other years were low flow years, with less than 135 mm. In particular, the rainfall amount of 67.9 mm in 2005 is the minimum rainfall amount, holding the lowest rainfall record over the past 59 years. During the experiment period, a total of 210 rainfall events were measured, accounting for 94.7% of the total rainfall amount (78.6 to 100%) and 82.7% of the number of rainfall events (66.7 to 100%) (Table S1).

**Figure 1:**
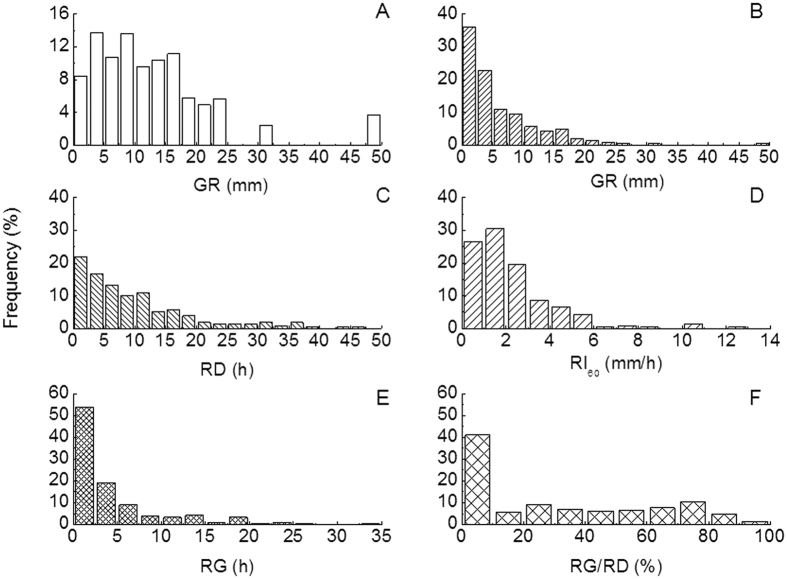
Frequency diagram of rainfall properties for 210 rainfall events during the experiment in 2004–2014 except for 2007. Rainfall properties contains the frequency of different rainfall amount to total rainfall amount (**A**), frequency of number of rainfall events to total number of rainfall events (**B**), frequency of number of rainfall durations occupied in total rainfall events (**C**), frequency of maximum stage rainfall intensity in 60 min occupied in total rainfall events (RI_60_, (**D**)), frequency of period of rainless gap within rainfall event occupied in total rainfall events (RG, (**E**)), and frequency of rainless gap to rainfall duration occupied in total rainfall events (RG/RD, (**F**)).

For the 210 rainfall events we measured during the 10-year experimental period, the average GR was 6.25 mm, ranging from 0.6 to 48.2 mm, in which <20 mm and <12.5 mm rainfall events occupied 83.4 and 56.1% of the total rainfall amounts, respectively. In addition, <5 mm and <10 mm rainfall events occupied 59.0 and 79.5% of the total rainfall number. The averaged RD was 9.85 hours, and ranged from 0.08 to 46.5 hours, and 51.9 and 83.8% of individual rainfall events had RD of less than 7.5 and 17.5 hours. The averaged maximum rainfall intensities, i.e. RI_10_(18.2 mm h^−1^), RI_30_(3.30 mm h^−1^) and RI_60_(2.16 mm h^−1^), showed decreasing intensity with increasing aggregation time, and were less than the average RI, at 1.56 mm h^−1^. Furthermore, the coefficients of variation (CV) of RI_10_(953%), RI_30_(91.9%) and RI_60_(87.9%) showed a decreasing trend with increasing aggregation time, compared to the CV of RI, at 209%. The averaged RG was 4.08 hours, and ranged from 0 to 32.7 hours, while 53.8% of individual rainfall events had RG of less than 2.5 hours. The averaged RG/RD was 30.4%, and ranged from 0 to 92.1%, of which less than 10% of RG/RD occupied 55.7% of rainfall events (Fig. 1 and Table S2).

**Figure 2:**
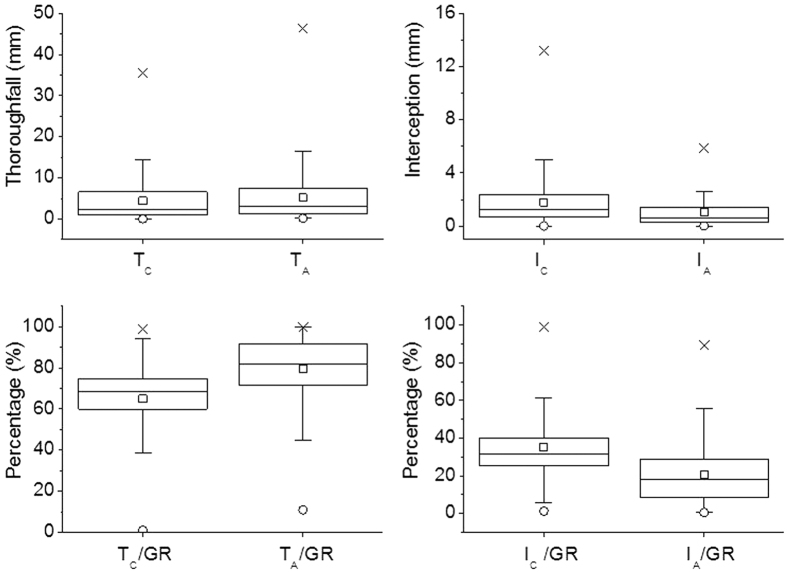
T_C_ and T_A_, I_C_ and I_A_, T_C_/GR and T_A_/GR, I_C_/GR and I_A_/GR with GR during experiment in 2004–2014 except for 2007. The symbols “×”, “◽” and “⚪” refer to the maximum, mean and minimum values, respectively.

During the experiment period, the average T_C_ and T_A_ were 4.39 (0.019–35.4 mm) and 5.13 mm (0.12–46.4 mm), respectively; average T_C_/GR and T_A_/GR were 70.9% (1.04–98.9%) and 82.9% (10.9–99.6%); average I_C_ and I_A_ were 1.80 (0.011–13.2 mm) and 1.05 mm (0.011–5.87 mm); and average I_C_/GR and I_A_/GR were 29.1% (1.15–99.0%) and 17.1% (0.421–89.1%) (Fig. 2).

### Relationships between throughfall and interception with rainfall properties

**Figure 3:**
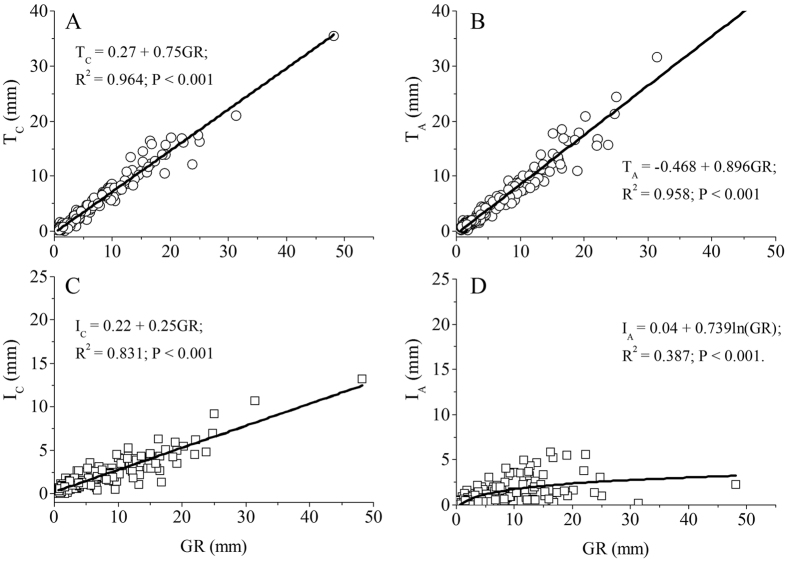
Observed (circle or square) and simulated (solid line) relationships between T_C_ and T_A_ with GR (**A**,**B**), and I_C_ and I_A_ with GR (**C**,**D**).

**Figure 4:**
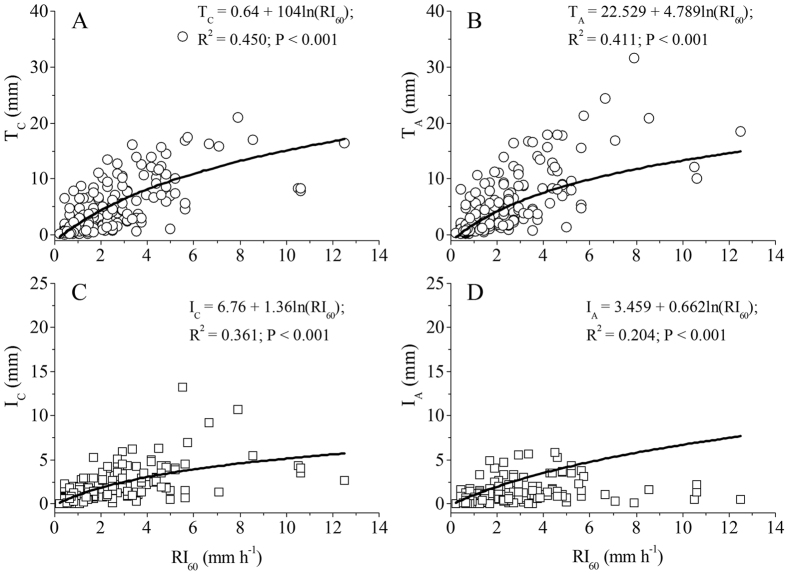
Observed (circle or square) and simulated (solid line) relationships between T_C_ and T_A_ with RI_60_ (**A**,**B**), and I_C_ and I_A_ with RI_60_ (**C**,**D**).

Both the linear and exponential models could very effectively describe the relationships between throughfall and interception with GR for *C. korshinskii* (T_C_ and I_C_), due to the fact that both models had very close R^2^ values and AIC scores, and the fitted models explained more than 80% of the variations. However, the best model to describe the relationship between T_A_ and GR (i.e. explained 95.8% of the variations) was linear. The relationship between I_A_ with GR was described well by the logarithmic model with the lowest AIC, yet explained only 38.7% of the variations, and was also described by exponential equation with the highest R^2^ of 0.693, but a higher AIC score (Fig. 3 and Table S3). The linear equation was the best model to describe the correlations of T_C_, T_A_, I_C_ and I_A_ with RD, and explained more than 12% of the variations (Table S3). The logarithmic model showed significant correlations of T_C_, T_A_, I_C_ and I_A_ with RI_60_ and explained more than 24% of the variations (Fig. 4 and Table S3). The relationships of T_C_, T_A_, I_C_ and I_A_ with RG could be described by a linear model, though the relationship between T_A_ and RG arrived at the margin significant level, and the fitted models only explained about 5% of the variations (Table S3). In addition, the best model to describe the relationships of T_C_, T_A_, I_C_ and I_A_ with RI_10_, RI_30_ and RI was logarithmic; however, all of them had lower R^2^ values and higher AIC scores than those between them and the RI_60_ (Table S3). At the same time, the relationships of the relative throughfall and interception (T_C_/GR, T_A_/GR, I_C_/GR and I_A_/GR) with GR, GD and RI_60_ were best fitted by the logarithmic model on the basis of R^2^ values and AIC scores (Table S3).

### Relationships between throughfall and interception with crown characteristics

The linear and logarithmic models showed marginally significant or significant correlations of T_C_ with H and BLAI, explained merely 1.4 and 0.3% of the variations, respectively. The relationships between T_A_ with H, BLAI, C_A_ and C_V_ were described significantly or marginally significantly by the linear model with the lowest AIC, but explained no more than 3% of the variations. For interception, the relationships between I_C_ with H, BLAI, C_A_ and C_V_ were described significantly or marginally significantly by the logarithmic model, but explained no more than 5% of the variations. The relationships between I_A_ with H, BLAI, C_A_ and C_V_ were described well by the exponential model, explained also no more than 5% of the variations. The relationships between T_A_/GR and I_A_/GR with H, BLAI, C_A_ and C_V_ were described well by the exponential model, explained no more than 6% of the variations. However, no significant relationships between T_A_/GR and I_C_/GR with crown characteristics were found (Table S4).

### Comprehensive analysis on effects of rainfall properties and crown characteristics on throughfall and interception

**Figure 5:**
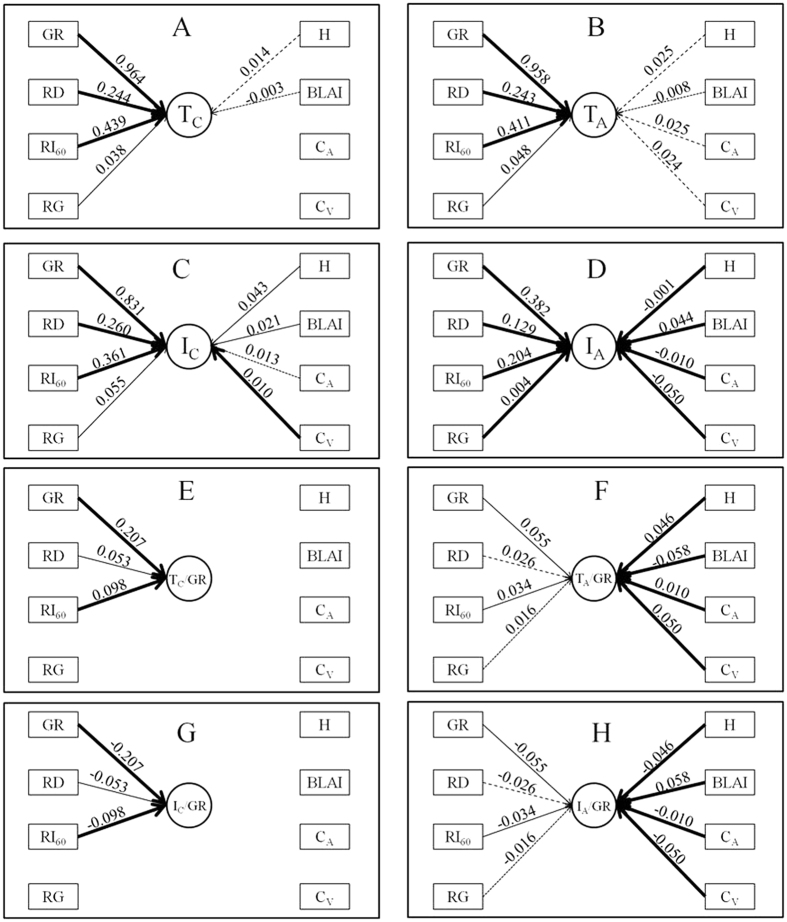
Comprehensive analysis on relationships between T_C_ (**A**), T_A_ (**B**), I_C_ (**C**), I_A_ (**D**), T_C_/GR (**E**), T_A_/GR (**F**), I_C_/GR (**G**) and I_A_/GR (**H**) with rainfall properties (GR, RD, RI_60_ and RG) and crown characteristics (H, BLAI, C_A_ and C_V_). The bold solid line means P < 0.001, solid line means P < 0.01, dashed line means P < 0.05, and dottted line means P < 0.1. The number above the line is R^2^ value from univariable regression analysis (details refer to Tables S3 and S4). “−” means negative relationship.

For throughfall and interception of two shrubs, the rainfall properties showed more importance than the crown characteristics. T_C_, T_A_, I_C_ and I_A_ can be explained by rainfall properties with a deceasing importance of GR, RI_60_, RD and RG using a univariable regression. However, the crown characteristics had no a certain order to explain them. H and BLAI were two crown characteristics to explain T_C_ with marginal significance. H and C_A_ were the most explained for T_A_, following by C_V_ and BLAI. I_C_ can be explained by crown characteristics with deceasing importance of H, C_V_, BLAI and C_A_ using a univariable regression. C_V_ was the most explained for I_A_, followed by BLAI, C_A_ and H (Fig. 5).

For relative throughfall and interception, the importance of rainfall properties and crown characteristics depended on shrub species. T_C_/GR and I_C_/GR can be only significantly explained by rainfall properties, GR, RI_60_ and RD showed a decreasing importance using a univariable regression. All of crown characteristics had extremely significant (P < 0.001) influence on T_A_/GR and I_A_/GR, with a deceasing importance of BLAI, C_V_, H and C_A_ using a univariable regression. However, rainfall properties except for GR had less importance on T_A_/GR and I_A_/GR compared to the crown characteristics (Fig. 5).

The stepwise regression analyses suggested the model on T_C_ only increased the R^2^ from 0.964 to 0.968 when the number of dependent variables increased from one (GR) to three (GR, GD, RI_60_ or BLAI), but the lowest AIC was −83 with variables of GR and RD. The model on T_A_ with the univariable of GR explained 84.3% of the variation with AIC was 242, and increased to 88.3% of the variation when RI_60_, RG and H were moved into the equation with AIC of 185. The model on I_C_ with the univariable of GR explained 83.1% of the variation with AIC of −167, and increased to 83.6% of the variation when H was moved into the equation without AIC d decrease. The model on I_A_ with the univariable of GR explained 69.3% of the variation with AIC of 165, and increased to 76.1% of the variation when H was moved into the equation with AIC of 111. The model on T_C_/GR and I_C_/GR with the univariable of GR explained 12.0% of the variation with AIC of 1063, and increased to 14.4% of the variation when C_V_ was moved into the equation without AIC decrease. The model on T_A_/GR and I_A_/GR with the univariable of H explained 37.7% of the variation with AIC of 1218, and increased to 49.5% of the variation when GR, RI_60_ and C_V_ were moved into the equation with AIC of 1180 (Table S5).

## Discussion

### Interception loss by sand-fixed shrubs

During the study period, the mean percentages of interception to gross rainfall values were 29.1% for *C. korshinskii* and 17.1% for *A. ordosica*. The values were slightly higher than those we measured from 2004 to 2006, with 27.0% for *C. korshinskii* and 15.0% for *A. ordosica*^11^. In addition, the canopy interception loss by revegetated shrubs was close to the values reported in other studies performed in arid and semi-arid regions, such as 17.8% in an afforested park land in a semiarid region of Iran^12^, with 21.7 and 20.7% for *Acacia farensiana* and *Prosopis laevigata* trees^30^; 27.9% for shrub in a semiarid climate zone in Mexico^31^; 26.8% for *Quercus rotundifolia* trees in a semiarid region of Spain^32^; and 13.0% for oak forests^33^ and 18.9% for semiarid *Tamaulipan thorn scrub*^34^.

The high rainfall interception loss by sand-stabilizing shrubs was mostly attributed to two causes, i.e. the frequency of small rainfall events, and the very high evaporation rate within a rainfall event. Our results showed that the rainfall events in the Shapotou region were characterized by small rainfall amounts (averaged 6.25 mm per event, and nearly 60 and 80% of rainfall events are less than 5 and 10 mm), low rainfall intensity (averaged 1.56 mm h^−1^) and long period of RG (accounting for 30% of the rainfall period). These rainfall properties determined that rainwater is more likely to be lost while wetting the crown surface and less likely to pass through the plant crown^20^. In addition, the ongoing evaporation during rain events significantly impacted interception loss, although we did not measure it during rain. Due to the fact that in dryland ongoing wet canopy evaporation rate can be very rapid, the evaporation rate was 2.51–3.31 mm h^−1^, as estimated by the Gash interception model^34^, and 5.0–8.0 mm h^−1^ estimated by the Rutter interception model^9^. Dunkerley^20^ showed that the evaporation from the wet foliage during rain proceeds at an average rate of 3.6 mm h^−1^ (from 2.06–5.76 mm h^−1^) in the Australian dryland. Clearly, these evaporation rates during rain were greater than the average RI in our study.

Stemflow is one of the most important components of rainfall redistribution. During 2010 and 2011 at our study site, Wang *et al*.^35^ observed stemflow accounted for 6.6% for *C. korshinskii* and 1.8% for *A. ordosica* of the individual gross rainfall, in 32 individual rainfall events with a total rainfall amount of 248.9 mm. In our study, interception is equal to rainfall minus throughfall, based on the assumption of minimum contribution from stemflow, which was not measured in our study. Although a potential overestimation of interception is implied in this assumption, it can be ignored, as shown in previous studies^36^,^37^. The main reason for this is the crown structures of the two desert shrubs, as well as the rainfall properties. The branches of the matured *A. ordosica* generally bend onto the ground, and those of *C. korshinskii* have multiple stems^11^. Neither of these two features favors the production of stemflow. In addition, West and Gifford^38^ suggested stemflow can be neglected in the interception loss calculation for shrubs with stem diameters of 10 to 20 mm. The basal stem diameters for the thickest stems of *A. ordosica* and *C. korshinskii* are on the upper edge of this range^11^,^35^. Furthermore, the small rain events in our experimental site may have generated little or no stemflow (e.g., Cecchi *et al*.^19^). Regardless of this, when the stemflows (6.6 and 1.8%) observed by Wang *et al*.^35^ were compared to the values of interception loss (29.1 and 17.1%) obtained in our study, the neglect of stemflow in our study resulted in slight overestimation of interception loss, and was acceptable.

### Effects of rainfall properties and crown characteristics on interception

Using regression analysis, we found rainfall properties have more contributions than crown characteristics on throughfall and interception of shrubs. GR, RI_60_, GD and RG to T_C_, T_A_, I_C_ and I_A_ showed a decreasing sequence on the basis of R^2^ values and AIC scores, indicating that their importance to the throughfall and interception of *C. korshinskii* and *A. ordosica* have the same order (Fig. 5). The stepwise regression analysis showed that the model on T_C_ with three variables were GR, RD, RI_60_ or BLAI, while the model on T_A_ with four variables were GR, RI_60_, RG and H. The models of I_C_ and I_A_ with two variables were GR and H. The difference may be related to the differences in species characteristics. The leaves of *C. korshinskii* are covered by dense, white, soft and wooly hairs^35^, but the leaves of *A. ordosica* secrete volatile oils^39^. Therefore, rain drops can easily persist on the leaves of *C. korshinskii* in comparison to those of *A. ordosica*, resulting in the RG having less importance to T_C_ than T_A_, while emphasizing the importance of RD to T_C_. In addition, the branches of *A. ordosica* often touch the ground, and rainwater can flow easily along the branches to the ground. Therefore, it is determined that a long period of RG during rain would be more beneficial to *A. ordosica* than *C. korshinskii*, as the canopy of *A. ordosica* can empty water faster than that of *C. korshinskii* during the same period of RG. In short, GR was first important factor to influence on throughfall and interception of two shrubs, other rainfall properties, especially RD and RG should also be considered specific to difference plant species when building throughfall models.

Verifying our primary hypothesis, RI_60_ was the secondary important rainfall property influencing the throughfall and interception of shrubs, indicating by either it alone explaining 20 to 45% variations of them, and it is always a significant factor in stepwise regression analyses of T_C_ and T_A_. In most cases, rainfall records are converted to an annual series of the maximum rainfall rate or depth for particular time intervals, such as 10, 30 and 60 min (RI_10_, RI_30_ and RI_60_) and average rainfall intensity (RI)^6^,^13^. It is widely recognized the meaning or significance of measures of RI varies with the integration or aggregation time used to define the rate. Our results showed that the maximum rainfall intensities have a decreasing intensity with increasing aggregation time (from RI_10_ of 18.2 mm h^−1^ to RI_60_ of 2.16 mm h^−1^), which is in accordance with previous observations (e.g., Dunkerley^6^,^13^). In addition, our results showed firstly that the CV values of the maximum rainfall intensities present a decreasing trend with increasing aggregation time, and that the RI_60_ has the minimum CV value of 87.9%. Its closed value to evaporation rate during rain (e.g. 2.0–8.0 mm h^−1^) and the fact that it had the lowest CV were possible reasons that the RI_60_, in comparison to other rainfall intensities (RI_10_, RI_30_ and RI), is the best representative intensity to interpret the throughfall and interception of shrubs. The events defined using large minimum inter-event times would have intervals of reduced rain rate, during which canopy drip and evaporation could partially empty the leaf and stem storages. Therefore, we believe the RI_60_ should be taken as an important parameter to establish interception and throughfall models.

Intra-event (or within-storm) RG refers to the fraction of no-rain periods during a rainfall event^40^. The RG at our study site was averaged 4.08 h, accounting for about 30% of RD, which was similar to the values reported by previous studies in arid and semi-arid regions, such as 33%^41^ and 14–50%^14^, but was smaller than 75% in South Carolina^42^. The study results verified our hypothesis RG has significant impact on the throughfall and interception of shrubs, although RG has low contribution to them using univariable regression analysis (0.4–8.9%). RG may either increase the time required for attaining the maximum canopy storage capacity^21^, or allowing wet plant canopies to partially dry and re-develop the canopy storage capacity^3^, resulting in increasing interception losses. These matters warrant great attention because, in coming years, changes in pattern of rainfall associated with global climate change may result in interception losses, thereby contributing to growing water stress in arid and semi-arid ecosystems.

In our study, the linear models can very effectively describe the relationship of I_C_ with GR. This relationship differs from the logarithmic correlation of I_A_ with GR in our study, as well as the exponential relationships between interception and GR obtained in previous studies in arid and semi-arid regions^3^,^11^,^37^. The interception by plants generally showed a rapidly increasing trend with GR below its maximum canopy storage capacity, and would slow down when beyond the value, thus the exponential or logarithmic other than linear model was mostly used to fit the relationships between interception and GR. The abnormal phenomenon is possibly related to the crown structure and rainfall properties. In comparison to the small crown volume and water-repellency leaves of *A. ordosica*, *C. korshinskii* has a large crown volume and water-absorption leaves (i.e. leaves covered by dense hairs), resulting *C. korshinskii* having a non-sensitive response to the low GR, low intensity and long period of RD of rainfall event characteristic of arid and semi-arid regions. In particular, the canopy water storage capacity cannot reach the maximum during the rainfall event with long-term RD with low intensity or long period of RG, thus interception increased linearly with GR in our study area. This highlighted the fact that the influence of RD and RG on interception is species-dependent, and the interception of *C. korshinskii* with large crown is easily affected by RD.

BLAI had a close relationship with canopy water storage capacities. Similar to previous studies^7^,^23^,^24^, our results showed that the BLAI of two shrubs has negative and positive relationships with throughfall and interception, respectively. For same species, the bigger canopies meant the larger surface area which impact significantly rainfall capture capacities^11^,^43^. Using a rainfall simulator with eight rainfall intensities (from 1.15 to 11.53 mm h^−1^), Wang *et al*.^43^ observed the canopy water storage capacities were average 0.22 and 0.30 mm for *A. ordosica* and *C. korshinskii*, respectively. Using data of three former years (2004–2006), we estimated that the canopy water storage capacities are 0.52 and 0.68 mm for *A. ordosica* and *C. korshinskii*, respectively^11^. Obviously, the values we estimated using field data were higher than those using simulation method by Wang *et al*. Also, using data of three former years, we estimated the maximum canopy water storage capacities of *A. ordosica* and *C. korshinskii* were 1.01 and 4.94 mm^11^. In the present study, giving the maximum GR in an individual rain event was 60 mm throughout almost 60 years of climatic records (1956~2014), we estimated the maximum canopy water storage capacities were 3.07 and 15 mm for *A. ordosica* and *C. korshinskii* using univariable models, which were obviously higher than those by three years of data though the relationship between GR and I_A_ was exponential. This indicated the results gained by short- and long-term monitors are very possibly contradictory, thus herein we emphasized long-term monitoring is very necessary to study the hydrological process.

For relative throughfall and interception, we found that two shrub species have different respondences to rainfall properties and crown characteristics. For *C. korshinskii*, the relative throughfall and interception are closely related to the rainfall properties, and the importance of GR, RI_60_ and GD on them showed a deceasing sequence using a univariable regression. Also, the stepwise regression analysis showed that the models on T_C_/GR and I_C_/GR with two variables were GR and C_V_. This was slightly different from our former three-year observations, i.e., the positively linear relationships between T_C_/GR and I_C_/GR with the C_V_ and BLAI^11^. The main reason for this may have been after being planted for 15 years, *C. korshinskii* growth reaches its peak and results in deep soil water degradation. After that time, *C. korshinskii* hardly grows and maintains its stable crown characteristics. However, for *A. ordosica* with high regeneration rate in sand-fixed dunes^44^, we found crown characteristics, rather than rainfall properties, have extremely significant influences on T_A_/GR and I_A_/GR using uni-or multi-variable regressions. The result was very different from our previous three-year observations, i.e., no significant relationships were found between them^11^. During the three-year observation (2004–2006), a juvenile individual was selected, which had small size, low BLAI and upward branches, and had little influence on the relative throughfall and interception^11^. After 2007, as the individual of *A. ordosica* grew in size, the branches of the mature ones often reached the ground, and rainwater flowed easily along the branches to the ground. Therefore, this led to the T_A_/GR and I_A_/GR having positive and negative relationships with the H and C_V_, respectively. Again, we highlighted long-term observation is very necessary to study the influences of change in crown characteristics on throughfall and interception.

## Conclusion

Rainfall properties, especially GR and RI_60_ during rain events, had more significant impact on the throughfall and interception of two desert shrubs than the crown characteristics. However, for relative throughfall and interception, the two shrubs have different respondences to rainfall properties and crown characteristics, while those of *C. korshinskii* were merely closely related to rainfall properties, and those of *A. ordosica* were more dependent on crown characteristics. Closed value to evaporation rate during rain and lowest CV, RI_60_, rather than other rainfall intensities, was the secondary rainfall property influencing on throughfall and interception. Indeed, the RD and RG during a rainfall event affected the interception and throughfall of the shrubs and changed the water balance on dry land, but the influences of RD and RG on them were species-dependent. Here we highlight long-term monitoring is very necessary to determine the relationships between throughfall and interception with crown characteristics.

## Additional Information

**How to cite this article**: Zhang, Z.-S. *et al*. Gross rainfall amount and maximum rainfall intensity in 60-minute influence on interception loss of shrubs: a 10-year observation in the Tengger Desert. *Sci. Rep*. **6**, 26030; doi: 10.1038/srep26030 (2016).

## Supplementary Material

Supplementary Information
