# Supplementary material for: Gross rainfall amount and maximum rainfall intensity in 60-minute influence on interception loss of shrubs: a 10-year observation in the Tengger Desert
Source: Sci Rep. 2016 May 17;6:26030. doi: 10.1038/srep26030 (PMC4869001; doi:10.1038/srep26030)
Supplement: Supplementary Information [file srep26030-s1.pdf]

Appendix

Title: Gross rainfall amount and maximum rainfall intensity in 60 minutes influence on interception loss of shrubs: a 10 years observation in the Tengger Desert

Authors: Zhi-Shan Zhang, Yang Zhao*, Xin-Rong Li, Lei Huang, Hui-Juan Tan

***Corresponding author. Tel.** +86 931 4967133.

E-mail：zhaoyang66@126.com (Y. Zhao)

Figure S1 Photo showing layouts of throughfall collecting cups for *C. korshinskii* (upper) and *A. ordosica* (lower). Photos were taken in July 2004.

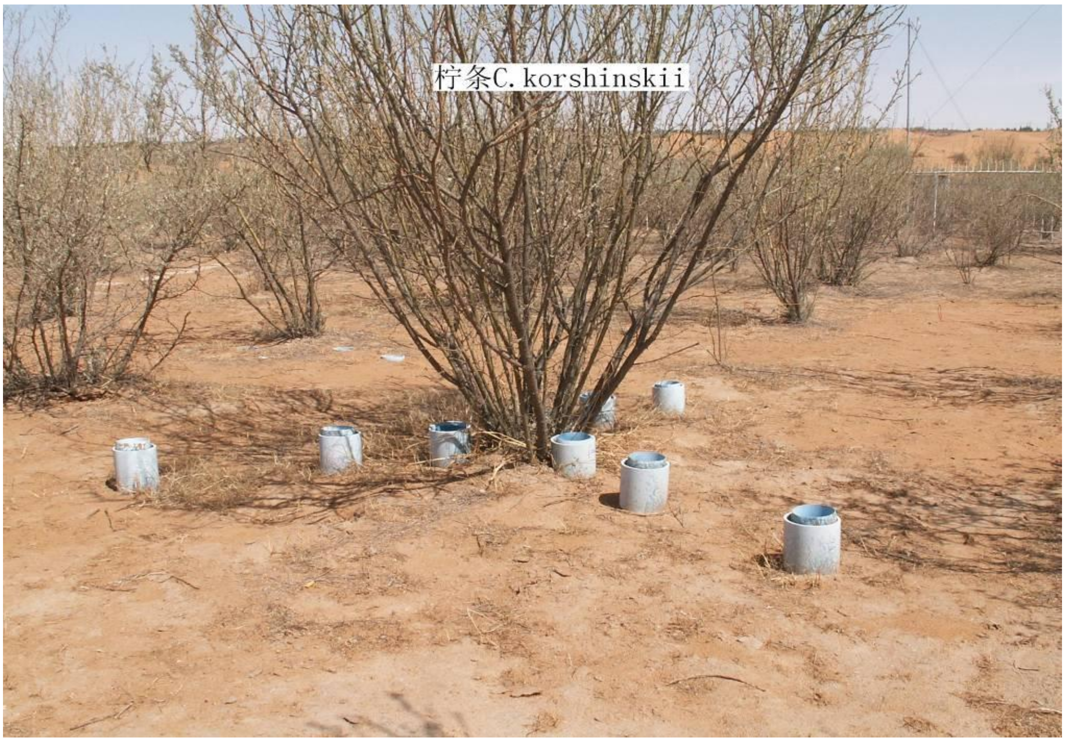

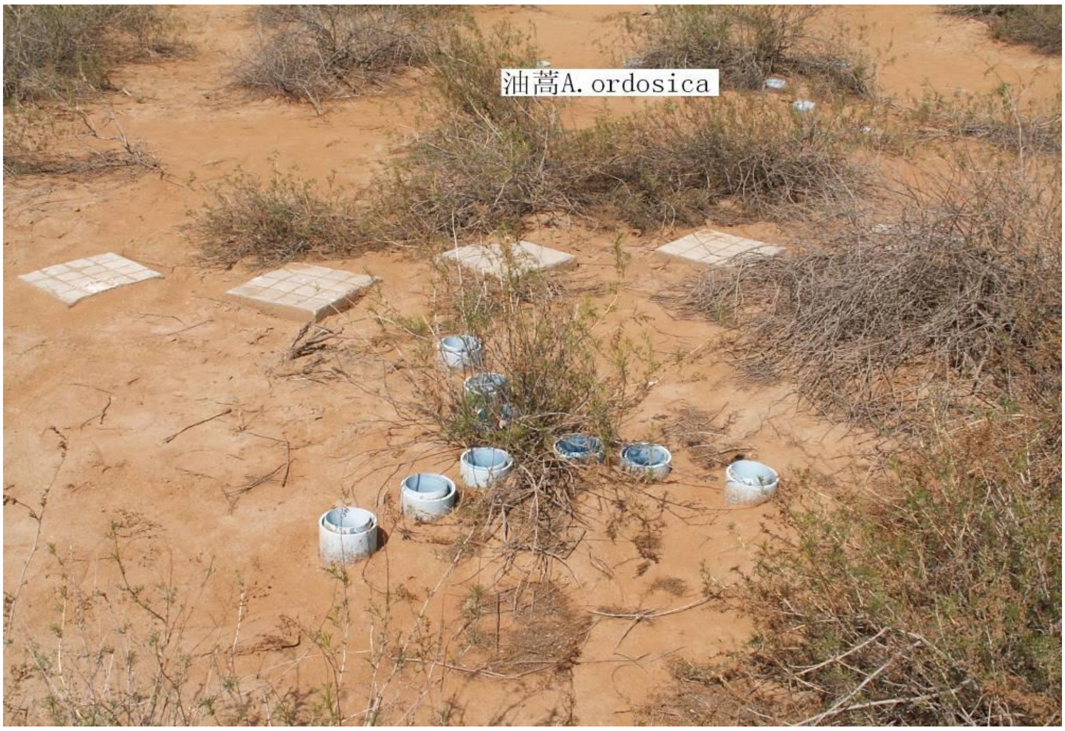

Tables S1 Total rainfall amount of the year (GRY), measuring rainfall amount of the year (M-GRY), percentage of measuring rainfall amount to total rainfall amount (M-GRY/GRY), number of rainfall events of year (N), number of measuring throughfall getting across the plant crown and interception by the plant crown (M-N), percentage of measuring throughfall and interception to total number of rainfall (M-N/N) events during experiment in 2004-2014 except for 2007.

Year	GRY mm	M-GRY mm	M-GRY/GRY %	N	M-N	M-N/N %
2004	106.0	102.2	96.4	24.0	20.0	83.3
2005	67.9	60.5	89.1	19.0	14.0	73.7
2006	89.3	87.8	98.3	19.0	15.0	78.9
2008	130.0	102.2	78.6	26.0	18.0	69.2
2009	120.4	120.4	100.0	23.0	23.0	100.0
2010	121.1	109.0	90.0	27.0	18.0	66.7
2011	186.5	182.8	98.0	25.0	23.0	92.0
2012	195.1	191.4	98.1	30.0	27.0	90.0
2013	133.7	131.7	98.5	24.0	22.0	91.7
2014	224.6	223.6	99.6	37.0	30.0	81.1
Mean	137.5	131.2	94.7	25.4	21.0	82.7

Tables S2 Descriptive statistic of rainfall properties for 210 rainfall events during the experiment in 2004-2014 except for 2007. GR: gross rainfall in an individual rain event; RD: rainfall duration in an individual rain event; RI_{10}, RI_{30} and RI_{60}: the maximum rainfall intensities in 10 min, 30 min and 60 min in an individual rainfall event; RI: mean rainfall intensity; RG: the period of rainless gap within rainfall event; RG/RD: percentage of rainless gap to rainfall duration.

Items	Mean	Standard Error	Min.	Med.	Max.	CV %
GR (mm)	6.25	0.451	0.600	3.80	48.2	104
RD (h)	9.85	0.657	0.08	7.22	46.5	96.5
RI_{10} (mm h^{-1})	18.2	2.11	1.20	4.20	25.2	953
RI_{30} (mm h^{-1})	3.30	0.240	0.42	2.40	20.4	91.9
RI_{60} (mm h^{-1})	2.16	0.120	0.18	1.62	12.0	87.9
RI (mm h^{-1})	1.56	0.240	0.06	0.66	24.8	209
RG (h)	4.08	0.405	0.00	1.80	32.7	143
RG/RD (%)	30.4	2.09	0.00	24.4	92.1	99.7

Table S3 Regression analyses of throughfall getting across the plant crown (T) and interception by the plant crown (I), percentage of throughfall to gross rainfall (T/GR), percentage of interception to gross rainfall (I/GR) with GR, RD, RI_{10}, RI_{30} and RI_{60}, RI, RG and RG/RD using linear Model: y = a + b*x; logarithmic Model: y = a + b*ln(x); exponential Model: y = a + b*exp^((-c)*x), respectively. Subscripts A and C represent *A. ordosica* and *C. korshinskii*, respectively. AIC: Akaike information criterion. √ indicates the best model on basis of highest R^2 value and lowest AIC score.

Independent variable	a	b	c	R^2	P	AIC	Note
Linear for T_C							
GR (mm)	0.27	0.75		0.964	0.000	-24.8	√
RD (min)	1.89	0.004		0.244	0.000	613.2	√
RI_{10} (mm min^{-1})	4.38	0.04		0.001	0.712	671.9	
RI_{30} (mm min^{-1})	1.22	57.1		0.350	0.000	581.5	
RI_{60} (mm min^{-1})	0.64	104		0.450	0.000	546.5	
RI (mm min^{-1})	4.16	8.93		0.010	0.000	670.0	
RG (min)	3.72	0.003		0.038	0.004	663.8	√

Independent variable	a	b	c	R^2	P	AIC	Note
RG/RD (%)	4.84	-0.02		0.009	0.185	669.0	
Logarithmic for T_C							
GR (mm)	-1.58	4.36		0.752	0.000	379.1	
RD (min)	-4.99	1.62		0.178	0.000	630.7	
RI_{10} (mm min^{-1})	11.8	2.89		0.279	0.000	603.3	√
RI_{30} (mm min^{-1})	17.2	4.02		0.400	0.000	564.9	√
RI_{60} (mm min^{-1})	19.3	4.11		0.439	0.000	550.7	√
RI (mm min^{-1})	9.97	1.24		0.089	0.000	652.5	√
Exponential for T_C							
GR (mm)	173.20	-173.62	0.005	0.965	0.000	-30.6	√
RD (min)	4.40	72610	1.46E+04	0.000	0.281	671.8	
RI_{10} (mm min^{-1})	-1170	1174	-3.70E-05	0.001	0.400	671.9	

Independent variable	a	b	c	R^2	P	AIC	Note
RI_{30} (mm min^{-1})	-35340	35340	-0.002	0.350	0.000	581.5	
RI_{60} (mm min^{-1})	-21550	21550	-0.005	0.450	0.000	546.5	
RI (mm min^{-1})	-3673	3677	-0.002	0.010	0.364	670.0	
RG (min)	4.51	-0.90	-189	0.005	0.008	374.2	
RG/RD (%)	-8824	8828	1.70E-06	0.009	0.262	669.0	
Linear for T_A							
GR (mm)	-0.468	0.896		0.958	0.000	82.290	√
RD (min)	2.121	0.005		0.243	0.000	691.843	√
RI_{10} (mm min^{-1})	5.115	0.053		0.001	0.711	750.140	
RI_{30} (mm min^{-1})	1.341	68.341		0.346	0.000	661.147	
RI_{60} (mm min^{-1})	0.639	124.241		0.443	0.000	627.244	
RI (mm min^{-1})	4.877	9.751		0.008	0.196	748.583	

Independent variable	a	b	c	R^2	P	AIC	Note
RG (min)	4.223	0.004		0.048	0.001	739.909	√
RG/RD (%)	5.560	-0.014		0.005	0.302	748.502	
Logarithmic for T_A							
GR (mm)	-1.769	5.033		0.692	0.000	503.027	
RD (min)	-5.869	1.900		0.168	0.000	711.456	
RI_{10} (mm min^{-1})	13.863	3.396		0.266	0.000	685.408	√
RI_{30} (mm min^{-1})	20.118	4.687		0.375	0.000	651.685	√
RI_{60} (mm min^{-1})	22.529	4.789		0.411	0.000	639.039	√
RI (mm min^{-1})	11.408	1.398		0.077	0.000	733.346	√
Exponential for T_A							
GR (mm)	30.347	-31.024	0.029	0.869	0.000	203.966	
RD (min)	4.129	69990.0	14120.0	0.006	0.546	630.650	

Independent variable	a	b	c	R^2	P	AIC	Note
RI_{10} (mm min^{-1})	-3070.000	3074.000	-0.000	0.002	0.102	630.575	
RI_{30} (mm min^{-1})	-35900.000	35900.000	-0.001	0.321	0.000	549.586	
RI_{60} (mm min^{-1})	-22270.000	22270.000	-0.004	0.430	0.000	512.760	
RI (mm min^{-1})	4.044	0.000	-73.845	0.049	0.112	620.451	
RG (min)	4.373	-0.844	-177.469	0.003	0.341	379.025	
RG/RD (%)	4.501	-1.043	1.116	0.013	0.223	627.591	
Linear for I_C							
GR (mm)	0.22	0.25		0.831	0.000	-123.6	√
RD (min)	0.86	0.002		0.260	0.000	186.8	√
RI_{10} (mm min^{-1})	1.80	0.01		0.000	0.754	250.0	
RI_{30} (mm min^{-1})	0.80	18.1		0.262	0.000	186.3	
RI_{60} (mm min^{-1})	0.59	33.5		0.350	0.000	159.6	

Independent variable	a	b	c	R^2	P	AIC	Note
RI (mm min^{-1})	1.79	0.49		0.000	0.832	250.0	
RG (min)	1.51	0.001		0.055	0.001	238.1	√
RG/RD (%)	1.86	-0.002		0.001	0.587	247.7	
Logarithmic for I_C							
GR (mm)	-0.16	1.43		0.607	0.000	53.8	
RD (min)	-1.64	0.59		0.178	0.000	208.7	
RI_{10} (mm min^{-1})	4.12	0.90		0.203	0.000	202.3	√
RI_{30} (mm min^{-1})	5.97	1.30		0.314	0.000	170.9	√
RI_{60} (mm min^{-1})	6.76	1.36		0.361	0.000	156.1	√
RI (mm min^{-1})	3.31	0.34		0.048	0.001	239.7	√
Exponential for I_C							
GR (mm)	3490	-3490	7.28E-05	0.831	0.000	-123.5	√

Independent variable	a	b	c	R^2	P	AIC	Note
RD (min)	1.81	28240	5.68E+03	0.000	0.675	250.0	
RI_{10} (mm min^{-1})	-457	458	-2.94E-05	0.000	0.786	250.0	
RI_{30} (mm min^{-1})	-12100	12100	-0.001	0.262	0.000	186.3	
RI_{60} (mm min^{-1})	-9093	9093	-0.004	0.350	0.000	159.3	
RI (mm min^{-1})	-250	252	-0.002	0.000	0.665	250.0	
RG (min)	2.14	-0.64	-83.5	0.001	0.432	-90.9	
RG/RD (%)	1.99	-0.54	9.17	0.021	0.041	243.6	√
Linear for I_A							
GR (mm)	0.429	0.100		0.308	0.000	-10.669	
RD (min)	0.625	0.001		0.129	0.000	37.361	√
RI_{10} (mm min^{-1})	1.052	0.005		0.000	0.869	66.757	
RI_{30} (mm min^{-1})	0.685	6.634		0.084	0.000	48.255	

Independent variable	a	b	c	R^2	P	AIC	Note
RI_{60} (mm min^{-1})	0.594	12.702		0.120	0.000	39.919	
RI (mm min^{-1})	1.061	-0.310		0.000	0.834	66.740	
RG (min)	0.994	0.000		0.005	0.291	65.658	
RG/RD (%)	1.130	-0.003		0.007	0.243	55.125	
Logarithmic for I_A							
GR (mm)	0.040	0.739		0.387	0.000	-35.847	√
RD (min)	-0.728	0.308		0.115	0.000	40.685	
RI_{10} (mm min^{-1})	2.051	0.388		0.090	0.000	46.993	√
RI_{30} (mm min^{-1})	3.038	0.621		0.170	0.000	27.594	√
RI_{60} (mm min^{-1})	3.459	0.662		0.204	0.000	18.942	√
RI (mm min^{-1})	1.841	0.176		0.032	0.010	60.034	√
Exponential for I_A							

Independent variable	a	b	c	R^2	P	AIC	Note
GR (mm)	16690.000	-16690.0	0.000	0.693	0.000	165.740	
RD (min)	2.017	29490.0	5933.0	0.000	0.432	413.505	
RI_{10} (mm min^{-1})	-396.343	398.361	0.000	0.000	0.551	413.494	
RI_{30} (mm min^{-1})	-10500.000	10500.0	-0.002	0.216	0.000	362.311	
RI_{60} (mm min^{-1})	-9235.000	9236.0	-0.005	0.270	0.000	347.529	
RI (mm min^{-1})	-335.320	337.347	0.001	0.000	0.661	413.493	
RG (min)	2.095	-0.517	-136.427	0.004	0.000	-27.201	√
RG/RD (%)	-3.485	4.984	-308.421	0.001	0.000	-60.501	√
Linear for T_C/GR							
GR (mm)	59.3	0.90		0.120	0.000	1161.1	
RD (min)	61.6	0.01		0.036	0.006	1180.3	
RI_{10} (mm min^{-1})	64.8	0.20		0.001	0.618	1187.7	

Independent variable	a	b	c	R^2	P	AIC	Note
RI_{30} (mm min^{-1})	60.2	85.3		0.067	0.000	1173.4	
RI_{60} (mm min^{-1})	59.4	151.3		0.082	0.000	1170.0	
RI (mm min^{-1})	64.6	11.2		0.001	0.601	1187.7	
RG (min)	64.5	0.00		0.001	0.594	1187.7	
RG/RD (%)	66.4	-0.05		0.008	0.188	1185.9	
Logarithmic for T_C/GR							
GR (mm)	54.2	7.80		0.207	0.000	1139.3	√
RD (min)	47.4	3.06		0.053	0.001	1176.5	√
RI_{10} (mm min^{-1})	79.1	5.51		0.087	0.000	1168.8	√
RI_{30} (mm min^{-1})	87.0	6.90		0.101	0.000	1165.6	√
RI_{60} (mm min^{-1})	89.0	6.64		0.098	0.000	1166.2	√
RI (mm min^{-1})	74.1	2.04		0.021	0.038	1183.6	√

Independent variable	a	b	c	R^2	P	AIC	Note
Exponential for T_C/GR							
GR (mm)	64.9	-2.82	50.4	0.000	0.982	1188.0	
RD (min)	64.9	45670	10030	0.000	0.761	1187.9	
RI_{10} (mm min^{-1})	-4462	4527	-0.000	0.001	0.650	1187.7	
RI_{30} (mm min^{-1})	-71720	71780	-0.001	0.067	0.021	1173.4	
RI_{60} (mm min^{-1})	-46580	46640	-0.003	0.082	0.017	1170.0	
RI (mm min^{-1})	64.8	0.00	-31.9	0.009	0.124	1186.0	
RG (min)	66.0	-2.82	-128200	0.000	0.766	1034.8	
RG/RD (%)	-27240	27300	0.000	0.008	0.231	1185.9	
Linear for T_A/GR							
GR (mm)	75.883	0.553		0.055	0.001	1136.416	
RD (min)	76.742	0.004		0.026	0.021	1142.044	√

Independent variable	a	b	c	R^2	P	AIC	Note
RI_{10} (mm min^{-1})	79.292	0.140		0.001	0.702	1148.091	
RI_{30} (mm min^{-1})	76.067	58.906		0.039	0.004	1139.967	
RI_{60} (mm min^{-1})	75.686	100.925		0.044	0.002	1138.794	
RI (mm min^{-1})	79.105	8.800		0.001	0.651	1148.032	
RG (min)	0.005	78.005		0.016	0.072	1144.953	
RG/RD (%)	79.522	-0.002		0.000	0.953	1146.781	
Logarithmic for T_A/GR							
GR (mm)	74.286	3.683		0.056	0.001	1136.209	√
RD (min)	69.336	1.715		0.021	0.038	1143.115	√
RI_{10} (mm min^{-1})	89.166	3.824		0.051	0.001	1137.326	√
RI_{30} (mm min^{-1})	91.186	3.707		0.035	0.006	1140.709	√
RI_{60} (mm min^{-1})	92.304	3.570		0.034	0.007	1140.898	√

Independent variable	a	b	c	R^2	P	AIC	Note
RI (mm min^{-1})	82.749	0.761		0.003	0.397	1147.514	
Exponential for T_A/GR							
GR (mm)	62.223	-16.514	38.450	0.000	0.317	1318.401	
RD (min)	62.293	90860.000	19310.000	0.000	0.392	1317.995	
RI_{10} (mm min^{-1})	-9816.000	9878.000	-0.000	0.005	0.297	1317.417	
RI_{30} (mm min^{-1})	-53260.000	53320.000	-0.001	0.021	0.294	1314.014	
RI_{60} (mm min^{-1})	-67590.000	67650.000	-0.002	0.032	0.222	1311.529	
RI (mm min^{-1})	63.867	-77.403	1059.000	0.042	0.292	1309.471	
RG (min)	20.140	36.621	-83.080	0.121	0.117	1139.849	
RG/RD (%)	-78.178	140.510	0.000	0.000	0.127	1311.529	
Linear for I_C/GR							
GR (mm)	40.72	-0.90		0.120	0.000	1161.1	

Independent variable	a	b	c	R^2	P	AIC	Note
RD (min)	38.39	-0.01		0.036	0.006	1180.3	
RI_{10} (mm min^{-1})	35.16	-0.20		0.001	0.618	1187.7	
RI_{30} (mm min^{-1})	39.83	-85.3		0.067	0.000	1173.4	
RI_{60} (mm min^{-1})	40.57	-151.3		0.082	0.000	1170.0	
RI (mm min^{-1})	35.39	-11.2		0.001	0.601	1187.7	
RG (min)	35.53	-0.00		0.001	0.594	1187.7	
RG/RD (%)	33.58	0.05		0.008	0.188	702.4	
Logarithmic for I_C/GR							
GR (mm)	45.8	-7.80		0.207	0.000	1139.3	√
RD (min)	52.6	-3.03		0.053	0.001	1176.5	√
RI_{10} (mm min^{-1})	20.9	-5.51		0.087	0.000	1168.8	√
RI_{30} (mm min^{-1})	13.0	-6.90		0.101	0.000	1165.6	√

Independent variable	a	b	c	R^2	P	AIC	Note
RI_{60} (mm min^{-1})	11.0	-6.64		0.098	0.000	1166.2	√
RI (mm min^{-1})	25.9	-2.04		0.021	0.038	1183.6	√
Exponential for I_C/GR							
GR (mm)	24.7	28.9	0.280	0.219	0.000	1136.0	
RD (min)	23.9	13.0	-89.3	0.004	0.621	1034.8	
RI_{10} (mm min^{-1})	27.9	25.3	18.9	0.102	0.020	1165.3	
RI_{30} (mm min^{-1})	66520	-66480	-0.001	0.067	0.022	1173.4	
RI_{60} (mm min^{-1})	22.3	23.8	21.4	0.105	0.000	1164.6	
RI (mm min^{-1})	35.2	-0.00	-32.0	0.009	0.441	1186.0	
RG (min)	23.9	13.0	-89.3	0.004	0.677	1034.8	
RG/RD (%)	34.0	3.00	158	0.007	0.532	1186.2	
Linear for I_A/GR							

Independent variable	a	b	c	R^2	P	AIC	Note
GR (mm)	24.117	-0.553		0.055	0.001	1136.416	√
RD (min)	23.258	-0.004		0.026	0.021	1142.044	√
RI_{10} (mm min^{-1})	20.708	-0.140		0.001	0.702	1148.091	
RI_{30} (mm min^{-1})	23.933	-58.906		0.039	0.004	1139.967	
RI_{60} (mm min^{-1})	24.314	-100.925		0.044	0.002	1138.794	
RI (mm min^{-1})	20.895	-8.800		0.001	0.651	1148.032	
RG (min)	21.995	-0.005		0.016	0.072	1144.953	√
RG/RD (%)	20.478	0.002		0.000	0.953	1146.781	
Logarithmic for I_A/GR							
GR (mm)	25.714	-3.683		0.056	0.001	1136.209	√
RD (min)	30.664	-1.715		0.021	0.038	1143.115	√
RI_{10} (mm min^{-1})	10.834	-3.824		0.051	0.001	1137.326	√

Independent variable	a	b	c	R^2	P	AIC	Note
RI_{30} (mm min^{-1})	8.814	-3.707		0.035	0.006	1140.709	√
RI_{60} (mm min^{-1})	7.696	-3.570		0.034	0.007	1140.898	√
RI (mm min^{-1})	17.251	-0.761		0.003	0.397	1147.514	
Exponential for I_A/GR							
GR (mm)	28.747	30.790	0.369	0.117	0.317	1292.219	
RD (min)	37.220	-99550.0	-21040.0	0.612	0.392	1585.256	
RI_{10} (mm min^{-1})	30.484	17.551	11.860	0.031	0.297	1311.747	
RI_{30} (mm min^{-1})	74310.000	-74270.0	0.000	0.021	0.294	1314.014	
RI_{60} (mm min^{-1})	29.645	25.587	46.793	0.065	0.222	1304.305	
RI (mm min^{-1})	-926.627	964.466	0.002	0.000	0.292	1318.395	
RG (min)	36.792	2.686	104900	0.003	0.117	1317.727	
RG/RD (%)	36.780	2.500	36.714	0.003	0.127	1317.542	

Table S4 Regression analyses of T_C, T_A, I_C, I_A, T_C/GR, T_A/GR, I_C/GR and I_A/GR with height, branch and leaf area index (BLAI), crown area and crown volume using linear Model: y = a + b*x; logarithmic Model: y = a + b*ln(x); exponential Model: y = a + b*exp^((-c)*x), respectively. AIC: Akaike information criterion. √ indicates the best model on basis of highest R^2 value and lowest AIC score.

	Independent variable	a	b	c	R^2	F	P	AIC	Note
Linear for T_C									
	Height m	-3.81	4.12		0.014	3.896	0.050	669	√
	BLAI m^2	6.77	-2.66		0.003	10.825	0.001	670	√
	Crown area	-0.32	2.03		0.002	1.334	0.249	671	
	Crown volume	2.09	0.54		0.002	1.340	0.248	671	
Logarithmic for T_C									
	Height m	-278.0	-91.16		0.009	3.010	0.084	669	
	BLAI m^2	4.58	-0.97		0.003	2.820	0.094	669	
	Crown area	-66.21	-27.24		0.003	1.163	0.288	671	

	Independent variable	a	b	c	R^2	F	P	AIC	Note
	Crown volume	-26.92	-10.60		0.003	1.610	0.219	670	
Exponential for T_C									
	Height m	175.7	179.7	0.02	0.009	0.625	0.430	94	
	BLAI m^2	4.45	0.00	-19.39	0.002	0.112	0.784	94	
	Crown area	4.37	0.00	0.00	0.010	0.039	0.843	95	
	Crown volume	4.37	300.7	0.00	0.010	0.028	0.868	95	
Linear for T_A									
	Height m	1.13	4.69		0.025	6.316	0.013	713	√
	BLAI m^2	6.42	-2.16		0.008	2.747	0.099	745	√
	Crown area	2.55	0.99		0.024	6.172	0.014	720	√
	Crown volume	3.32	6.88		0.025	6.469	0.012	716	√
Logarithmic for T_A									

	Independent variable	a	b	c	R^2	F	P	AIC	Note
	Height m	-475.9	-141.1		0.020	0.119	0.730	631	
	BLAI m^2	4.51	0.84		0.000	1.680	0.192	629	
	Crown area	-588.5	-123.1		0.019	0.079	0.779	631	
	Crown volume	-351.4	-123.4		0.021	0.098	0.775	631	
Exponential for T_A									
	Height m	5.11	0.00	39066	0.010	0.219	0.730	166	
	BLAI m^2	5.09	0.00	0.00	0.010	0.299	0.742	165	
	Crown area	1458	1455.6	0.00	0.019	0.899	0.642	145	
	Crown volume	3192	3189.6	0.00	0.021	0.199	0.442	112	
Linear for I_C									
	Height m	-3.25	2.54		0.047	11.455	0.001	645	
	BLAI m^2	1.02	0.77		0.025	6.337	0.013	660	

	Independent variable	a	b	c	R^2	F	P	AIC	Note
	Crown area	-1.08	1.25		0.013	3.793	0.053	668	√
	Crown volume	0.35	0.34		0.014	4.066	0.045	667	
Logarithmic for I_C									
	Height m	-201.2	-63.1		0.043	9.54	0.002	33	√
	BLAI m^2	1.72	-0.8		0.021	7.18	0.008	35	√
	Crown area	-162.8	-45.6		0.008	3.080	0.081	38	√
	Crown volume	-187.4	-39.9		0.010	4.770	0.000	36	√
Exponential for I_C									
	Height m	1.66	0.00	0.74	0.015	2.000	0.159	10	
	BLAI m^2	0.98	0.00	193.8	0.219	0.460	0.498	12	
	Crown area	1.80	0.00	0.00	0.010	0.657	0.419	13	
	Crown volume	1.80	0.00	0.00	0.010	0.969	0.326	11	

	Independent variable	a	b	c	R^2	F	P	AIC	Note
Linear for I_A									
	Height m	0.94	0.13		0.004	0.130	0.719	67	
	BLAI m^2	0.57	0.79		0.040	9.727	0.002	58	
	Crown area	0.97	0.03		0.004	0.137	0.712	67	
	Crown volume	1.01	0.15		0.004	0.075	0.785	67	
Logarithmic for I_A									
	Height m	1.15	-0.08		0.006	33.12	0.000	382	
	BLAI m^2	1.15	-0.08		0.006	10.11	0.002	403	
	Crown area	1.06	-0.08		0.002	32.59	0.000	383	
	Crown volume	1.21	-0.07		0.003	32.60	0.000	383	
Exponential for I_A									
	Height m	1.11	71.8	10.5	0.001	41.8	0.000	15	√

	Independent variable	a	b	c	R^2	F	P	AIC	Note
	BLAI m^2	1.71	1.48	1.49	0.044	19.3	0.000	35	√
	Crown area	1.05	0.00	658	0.010	46.3	0.000	11	√
	Crown volume	1.05	0.00	2870	0.050	44.8	0.000	-100	√
Linear for T_C/GR									
	Height m	80.0	-7.69		0.001	1.134	0.288	1190	
	BLAI m^2	66.4	-1.64		0.003	0.320	0.572	1191	
	Crown area	82.4	-7.65		0.003	1.605	0.207	1189	
	Crown volume	74.1	-2.22		0.004	1.913	0.168	1190	
Logarithmic for T_C/GR									
	Height m	1123.2	290.7		0.004	0.623	0.477	1187	
	BLAI m^2	64.4	0.56		0.002	0.552	0.489	1187	
	Crown area	1445.5	358.3		0.002	1.172	0.850	1187	

	Independent variable	a	b	c	R^2	F	P	AIC	Note
	Crown volume	1925.6	364.1		0.001	0.172	0.280	1187	
Exponential for T_C/GR									
	Height m	65.5	0.00	-30.18	0.038	0.480	0.489	-249	
	BLAI m^2	65.0	0.00	-27.33	0.006	0.177	0.674	-248	
	Crown area	64.7	0.00	3929270	0.010	1.402	0.238	-250	
	Crown volume	64.7	0.00	2691	0.010	1.420	0.238	-250	
Linear for T_A/GR									
	Height m	65.1	16.68		0.050	12.109	0.001	1062	
	BLAI m^2	94.8	-25.66		0.067	1.546	0.000	1087	
	Crown area	69.66	3.69		0.054	12.983	0.000	1056	
	Crown volume	72.6	25.16		0.055	13.165	0.000	1049	
Logarithmic for T_A/GR									

	Independent variable	a	b	c	R^2	F	P	AIC	Note
	Height m	-1767.4	-532.71		0.045	112.0	0.000	1228	
	BLAI m^2	-1767.4	-532.71		0.045	15.7	0.000	1303	
	Crown area	-2023	-439.80		0.049	108.0	0.000	1231	
	Crown volume	-1035	-402.07		0.050	120.0	0.000	1232	
Exponential for T_A/GR									
	Height m	2733.9	2668	0.01	0.046	113.0	0.000	-209	√
	BLAI m^2	86.3	1.16	-2.39	0.058	30.0	0.000	-114	√
	Crown area	79.2	0.00	0.001	0.010	120.0	0.000	-214	√
	Crown volume	9697	9624	0.001	0.050	120.0	0.000	0	√
Linear for I_C/GR									
	Height m	19.97	7.69		0.001	1.134	0.288	1190	
	BLAI m^2	33.57	1.64		0.003	0.320	0.572	1191	

	Independent variable	a	b	c	R^2	F	P	AIC	Note
	Crown area	17.58	7.65		0.003	1.605	0.207	1189	
	Crown volume	25.91	2.22		0.004	1.913	0.168	1189	
Logarithmic for I_C/GR									
	Height m	-1023.27	-290.77		0.004	0.623	0.477	1187	
	BLAI m^2	35.55	-0.56		0.002	0.552	0.489	1187	
	Crown area	-1345.56	-358.31		0.002	1.172	0.850	1187	
	Crown volume	-1825.67	-364.19		0.001	0.172	0.280	1187	
Exponential for I_C/GR									
	Height m	35.23	0.00	244.03	0.038	0.480	0.489	-249	
	BLAI m^2	35.23	14182	115900	0.006	0.177	0.674	-248	
	Crown area	35.66	0.00	11.95	0.010	1.402	0.238	-250	
	Crown volume	35.65	561118	3.89	0.010	1.420	0.238	-250	

	Independent variable	a	b	c	R^2	F	P	AIC	Note
Linear for I_A/GR									
	Height m	34.96	-16.68		0.050	12.109	0.001	1141	
	BLAI m^2	5.18	25.66		0.067	77.368	0.000	1087	
	Crown area	30.34	-3.69		0.054	12.983	0.000	1140	
	Crown volume	27.35	-25.16		0.055	13.165	0.000	1140	
Logarithmic for I_A/GR									
	Height m	1867.41	532.71		0.045	112.0	0.000	1228	
	BLAI m^2	1867.41	532.71		0.045	15.7	0.000	1303	
	Crown area	2123	439.80		0.049	108.0	0.000	1231	
	Crown volume	1135.40	402.07		0.050	120.0	0.000	1232	
Exponential for I_A/GR									
	Height m	20.82	1931.83	2645	0.046	113.0	0.000	-209	√

Independent variable	a	b	c	R^2	F	P	AIC	Note
BLAI m^2	30294	30289	0.001	0.058	30.0	0.000	-114	√
Crown area	21.02	0.001	28.18	0.010	120.0	0.000	-214	√
Crown volume	20.82	0.001	14008	0.050	120.0	0.000	-220	√

Table S5 Stepwise regression analyses of T_C, T_A, I_C, I_A, T_C/GR, T_A/GR, I_C/GR and I_C/GR with GR, RD, RI_{60}, RG, height(H), BLAI, crown area (C_A) and crown volume (C_V) using Linear Model: y = a + b*GR + c*RD + d*RI_{60} + e*RG + f*H + g*BLAI + h*C_A+ I*C_V. SC means standardized coefficients, √ indicates the best model on basis of highest R^2 value and lowest AIC score.

Variables	a	b (sc)	c (sc)	d (sc)	e (sc)	f (sc)	g (sc)	h (sc)	i (sc)	R^2	P	AIC	Note
T_C (mm)	-0.268	0.746 (0.982)								0.964	0.000	-72	
T_C (mm)	-0.127	0.769 (1.01)	0.0002 (-0.056)							0.966	0.000	-83	√
T_C (mm)	0.265	0.774 (1.01)	0.0002 (-0.057)				-0.413 (-0.034)			0.967	0.000	-43	
T_C (mm)	0.156	0.748 (0.983)	0.0002 (-0.044)	-0.442 (-0.036)						0.968	0.000	-48	

T_A (mm)	0.171	0.632							0.843	0.000	242	
		(0.918)										
T_A (mm)	2.98	0.652				-3.458			0.870	0.000	230	
		(0.944)				(-0.167)						
T_A (mm)	3.06	0.667			-0.001	-3.334			0.878	0.000	224	
		(0.966)			(-0.091)	(-0.161)						
T_A (mm)	2.92	0.62		14.093	-0.001	-3.476			0.883	0.000	185	√
		(0.0898)		(0.100)	(-0.079)	(-6.845)						
I_C (mm)	0.217	0.254							0.831	0.000	-167	√
		(0.912)										
I_C (mm)	-1.55	0.25				0.907			0.836	0.000	-130	
		(0.899)				(0.081)						
I_A (mm)	-0.124	0.342							0.693	0.000	165	

		(0.833)						
I_A (mm)	-2.79	0.323	3.288		0.761		111	√
		(0.791)	(0.267)					
T_C/GR (%)	59.2	0.901			0.120	0.000	1063	√
		(0.347)						
T_C/GR (%)	68.29	0.953		-18.43	0.144	0.000	1157	
		(0.366)		(-0.154)				
T_A/GR (%)	117.7		-65.57		0.377	0.000	1218	
			(-0.614)					
T_A/GR (%)	115.1	1.088	-70.58		0.469	0.000	1187	
		(0.307)	(-0.661)					
T_A/GR (%)	102.1	1.1	-40.9	-47.129	0.485	0.000	1182	
		(0.31)	(-0.383)	(-0.305)				

T_A/GR (%)	102.1	0.798		95.02	-43.7	-43.898	0.495	0.000	1180	√
		(0.225)		(0.132)	(-0.41)	(-0.284)				
I_C/GR (%)	40.7	-0.901					0.120	0.000	1063	√
		(-0.347)								
I_C/GR (%)	31.7	-0.953				18.43	0.144	0.000	1157	
		(-0.366)				(0.154)				
I_A/GR (%)	-17.70				65.57		0.377	0.000	1218	
					(0.614)					
I_A/GR (%)	-15.14	-1.08			70.587		0.469	0.000	1187	
		(-0.307)			(0.661)					
I_A/GR (%)	-2.136	-1.1			40.899	47.129	0.485	0.000	1182	
		(-0.31)			(0.383)	(0.305)				
I_A/GR (%)	-2.16	-0.79	-95.02		43.779	43.898	0.495	0.000	1180	√

	(-0.225)	(-0.132)	(0.41)	(0.284)	
